# Rolipram and Electrical Stimulation Synergistically Promote Neuronal Differentiation of Adipose-derived Stromal Cells: an in Vitro Study

**DOI:** 10.1007/s12015-025-10925-5

**Published:** 2025-06-26

**Authors:** Milad Rahimzadegan, Alireza Soltani Khaboushan, Somayeh Niknazar, Mohammadhosein Ghahremani, Hamid Akbari Javar, Omid Sabzevari, Zahra Hassannejad

**Affiliations:** 1 Functional Neurosurgery Research Center, Shohada Tajrish Comprehensive Neurosurgical Center of Excellence, Shahid Beheshti University of Medical Sciences, Tehran, Iran; 2 Department of Pharmacology and Toxicology, School of Pharmacy, Tehran University of Medical Sciences, Tehran, Iran; 3 Department of Neurosurgery, Tehran University of Medical Sciences, Tehran, Iran; 4 Pediatric Urology and Regenerative Medicine Research Center, Gene, Cell and Tissue Research Institute, Tehran University of Medical Sciences, Tehran, Iran; 5 Students’ Scientific Research Center, Tehran University of Medical Sciences, Tehran, Iran; 6 Sina Trauma and Surgery Research Center, Tehran University of Medical Sciences, Tehran, Iran; 7 Department of Chemical and Environmental Engineering, University of Nottingham, Nottingham, NG7 2RD UK; 8 Biodiscovery Institute, University of Nottingham, Nottingham, UK; 9 Department of Toxicology and Pharmacology, Faculty of Pharmacy, Tehran University of Medical Sciences, Tehran, 141556451 Iran

**Keywords:** Nerve regeneration, Rolipram, Electrical stimulation, Conductive scaffold, Gold nanoparticles, Electrospun nanofibers

## Abstract

This study investigates gold-decorated polycaprolactone/chitosan nanofibers as conductive scaffolds for promoting neuronal differentiation of adipose-derived mesenchymal stromal cells (ADSCs) harvested from the dorsal interscapular region of Wistar rats under electrical stimulation (ES) with optimal rolipram concentrations. The scaffold was fabricated through electrospinning and in situ synthesis of gold nanoparticles (AuNPs). Morphology and AuNPs distribution were evaluated using a Field Emission Scanning Electron Microscope (FE-SEM) and energy dispersive X-ray spectroscopy (EDX). Rolipram, known to increase neuronal cyclic adenosine monophosphate (cAMP) activity and reduce inflammation, was loaded into the scaffolds using alginate hydrogel. The scaffolds were subjected to a release study and tests for ADSC proliferation and differentiation into neuron-like cells. Immunostaining of β-Tubulin III and MAP2 was used to assess the effect of ES, alone and in combination with rolipram, on the efficacy of neuronal differentiation of ADSCs. The distribution of AuNPs was uniform within the scaffolds with an electrical conductivity of 0.12 S.cm^−1^. Rolipram significantly improved the development of neurons from ADSCs, and this effect was more prominent at higher concentrations (1 and 5 µM). The study revealed that using an electrical density of 100 mV/mm, in combination with 5 µM rolipram and conductive scaffolds, led to a significant increase in the percentage of MAP2 and β-Tubulin III positive cells and the neuronal differentiation of ADSCs, with further elevation of cAMP levels compared to using 5 µM rolipram without ES. We found that combining rolipram and electrical stimulation at optimized doses and voltages can enhance nerve regeneration applications.

**Graphical Abstract:**

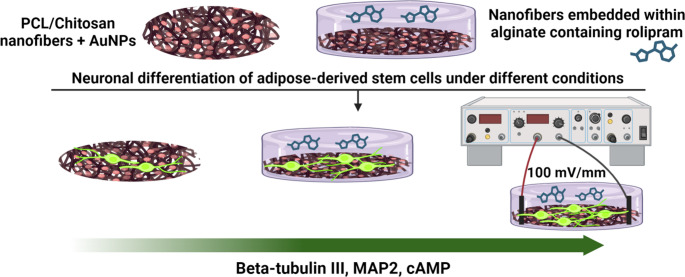

**Supplementary Information:**

The online version contains supplementary material available at 10.1007/s12015-025-10925-5.

## Introduction

The central nervous system (CNS), comprising the brain and spinal cord, has a limited capacity to regenerate after injury or disease [1, 2], with traumatic spinal cord injuries (TSCI) being the most common damage that results in cell death and axon degeneration, leading to the loss of sensory and motor functions [3, 4]. Currently, medical treatment for TSCI mainly focuses on stabilizing the damaged vertebrae, preventing the development of secondary injuries, and controlling inflammation [5]. However, tissue engineering has emerged as an alternative, promising therapeutic approach [6, 7]. Neural tissue engineering requires scaffolds that exhibit biocompatibility, optimized porosity and pore size for cell infiltration and angiogenesis, and controlled biodegradation [8, 9].

Electrospinning techniques have been employed to fabricate nanofibrous scaffolds from natural and synthetic polymers, such as polycaprolactone (PCL) and chitosan, which mimic the extracellular matrix of native tissue and provide a high surface area-to-volume ratio [10–12]. Chitosan is a natural polymer known for its non-toxic, biodegradable, and biocompatible properties. However, it has limited solubility and low mechanical strength in aqueous solutions [13]. There is a growing interest in creating three-dimensional (3D) hybrid scaffolds by combining natural and synthetic polymers to enhance their advantages and overcome their limitations. PCL is a biodegradable polyester that has been approved by the Food and Drug Administration (FDA). When PCL is blended with chitosan, it enhances the processing and mechanical properties of chitosan while addressing the hydrophilic and biological limitations of PCL. Therefore, the blend of PCL and chitosan could be an appropriate option for creating electrospun nanofibrous scaffolds.

The presence of electrical conductivity has a positive impact on cell proliferation and differentiation [14, 15], and different kinds of conductive scaffolds have been explored to enhance nerve regeneration [16, 17]. Incorporating AuNPs to induce electrical conductivity in polymeric scaffolds is of interest due to the unique electronic and optical properties of gold nanoparticles (AuNPs), and also their excellent biocompatibility. AuNPs have been utilized in various biomedical applications such as photothermal therapies, drug delivery systems, and also as detection/imaging agents [18–20]. Their ease of fabrication, nano-scale size, and capability to attach chemical agents to their surface render them suitable for biomedical applications [21]. Our previous works have shown that conductive PCL/chitosan nanofibrous scaffolds decorated with AuNPs have the potential to enhance both Schwann cell and fibroblast attachment and proliferation, as well as neuronal differentiation of mesenchymal stromal cells (MSCs) [22–25].

Adipose-derived mesenchymal stromal cells (ADSCs) are a valuable source of adult stromal cells for tissue engineering applications, because they are abundant and easily accessible. Large quantities of ADSCs can be acquired under local anesthesia with minimal discomfort. Additionally, ADSCs proliferate at a higher rate than bone marrow-derived stromal cells [26, 27].

Several mechanisms have been proposed for the effect of ES on neuronal differentiation and regeneration, including increased expression of neurotrophic factors such as brain-derived neurotrophic factor (BDNF) [28], increased axon transport [29], upregulation of regeneration-associated genes [30], as well as increased neurite outgrowth by mechanisms involving Ca^2+^ signaling and cyclic adenosine monophosphate (cAMP) [30]. Recent studies have shown that an increase in intracellular cAMP levels can promote axon regeneration [31], neuronal repair [32], and neuronal differentiation [33], while decreased intracellular cAMP levels are associated with a loss of neuronal regeneration [34]. The cAMP-hydrolyzing enzyme, phosphodiesterase-4 (PDE4), a key regulator of intracellular cAMP, hydrolyzes cAMP and reduces its intracellular concentration. Rolipram, a PDE4 inhibitor, enhances neuronal differentiation by modulating intracellular cAMP/CREB signaling pathways. By inhibiting PDE4, rolipram increases intracellular levels of cAMP. This elevation in cAMP, achieved through the inhibition of its hydrolysis, has been shown to promote axonal growth in the spinal cord when rolipram is administered via mini-pump infusion [35, 36].

In this study, our aim was to investigate the synergistic effect of rolipram and electrical stimulation on the intracellular level of cAMP and its effect on neuronal differentiation of ADSCs seeded on nanofibrous conductive scaffolds. We hypothesize that combining rolipram and electrical stimulation will increase intracellular cAMP levels and promote more robust neuronal differentiation of ADSCs compared to either treatment alone. These findings could have important implications for developing effective strategies for nerve tissue regeneration.

## Materials and Methods

### Materials

Polycaprolactone (PCL, MW = 80,000) and medium molecular weight chitosan (MW = 200,000–300,000) were purchased from Sigma-Aldrich (Germany). Formic acid (98–100%), acetic acid (100%), Gold-III-chloride trihydrate (HAuCl_4_.3H_2_O, ≥ 49% Au basis), ammonium hydroxide, sodium hydroxide, potassium carbonate, tetrakis (hydroxymethyl) phosphonium chloride (THPC) solution (80% in H_2_O), formaldehyde solution (H_2_CO, 37%), and absolute ethanol were acquired from Sigma-Aldrich (Darmstadt, Hesse, Germany). Phosphate Buffered Saline (PBS), 4′,6-diamidino-2-phenylindole (DAPI), 3-(4,5-dimethylthiazol-2-yl)−2,5-diphenyl-2 H-tetrazolium bromide (MTT, Sigma-Aldrich, Darmstadt, Hesse, Germany), Triton™X-100, and dimethyl sulfoxide (DMSO) were also obtained from Merck (Berlin, Germany). Dulbecco’s Modified Eagle Medium: Nutrient Mixture F-12 (DMEM/F-12), Trypsin-EDTA, fetal bovine serum (FBS), and penicillin-streptomycin solution were purchased from Gibco™ (Thermofisher Scientific, Waltham, MA, USA). Deionized water (18MX) was provided by a Milli-Q system (MilliporeSigma, Burlington, MA, USA). In addition, rolipram was acquired from Cayman (Ann Arbor, MI, USA), while the polyclonal antibodies against β-Tubulin III and MAP2 were obtained from Invitrogen (Thermo Fisher Scientific, Waltham, MA, USA). The cAMP assay kit (ab65355) was purchased from Abcam (Cambridge, MA, USA). All chemicals were used as received.

### Isolation and Characterization of Adipose-derived Mesenchymal Stromal Cells

All procedures were performed in accordance with the Ethics Guidelines of the Tehran University of Medical Sciences (TUMS). Approval was granted by the Ethics Committee of TUMS (IR.TUMS.CHMC.REC.1397.007). The ARRIVE guidelines (Animal Research: Reporting of In Vivo Experiments) were followed to report animal experiments. Adult Wistar rats were sacrificed (*n* = 2) by overdose with 300 mg/kg ketamine (10%; Medistar, Ascheberg, Germany) and 30 mg/kg Xylazine (2%; Riemser, Greifswald, Germany). The adipose tissue was harvested from the dorsal interscapular region of rats. Rats were provided by the Sina Trauma and Surgery Research Center. For cell isolation, the adipose tissues were washed three times with PBS, shredded into very fine pieces, and then digested using collagenase enzyme (I) for one hour at 37 °C. The enzyme activity was then neutralized by DMEM/F12 + 10% FBS cell culture medium. The resulting cell suspension was centrifuged, and ADSCs were obtained by removing the supernatant and adding DMEM/F12 + 10% FBS medium containing 1% penicillin and streptomycin. The isolated cells exhibited spindle-shaped, fibroblast-like morphology and plastic adherence, as evidenced by their attachment to culture flasks during passaging. The cell surface markers, including CD34, CD90, and CD105, were assessed at passage 3, using the flow cytometry technique to confirm the lineage of the extracted mesenchymal cells. Adipogenic and osteogenic differentiation were induced using protocols previously established in our research center [37, 38]. Adipogenesis was confirmed by Oil Red O staining following induction with dexamethasone and indomethacin. Osteogenesis was validated by Alizarin Red staining after treatment with ascorbic acid 2-phosphate, dexamethasone, and β-glycerol phosphate. Chondrogenic differentiation was confirmed by Alcian Blue staining for glycosaminoglycan detection [37, 38]. A comprehensive description of the staining process can be found in Supplementary File 1.

### Fabrication and Characterization of Nanofibrous Conductive Scaffolds

The nanofibrous scaffolds were fabricated using a method adapted from a previous approach [23]. The electrospinning process was conducted using a Dual Pump Electrospinning Machine (Side by Side Electroris^®^) at room temperature. The polymeric solution was prepared overnight by dissolving 0.3 g PCL and 0.03 g chitosan in 3 mL of acetic acid: formic acid solvent system (volume ratio of 7:3). Then, 400 µL of HAuCl_4_ in acetic acid (5 wt%) was added to the prepared polymeric solution. A high voltage of 15–18 KV was applied with a fixed needle tip-to-collector distance of 10 cm and a constant flow rate of 0.2 mL/h. The nanofibers were collected on an aluminum foil and then stabilized through immersion in a 5 M aqueous solution of ammonium hydroxide for 15 min and rinsed three times with distilled water [22].

**Fig. 1:**
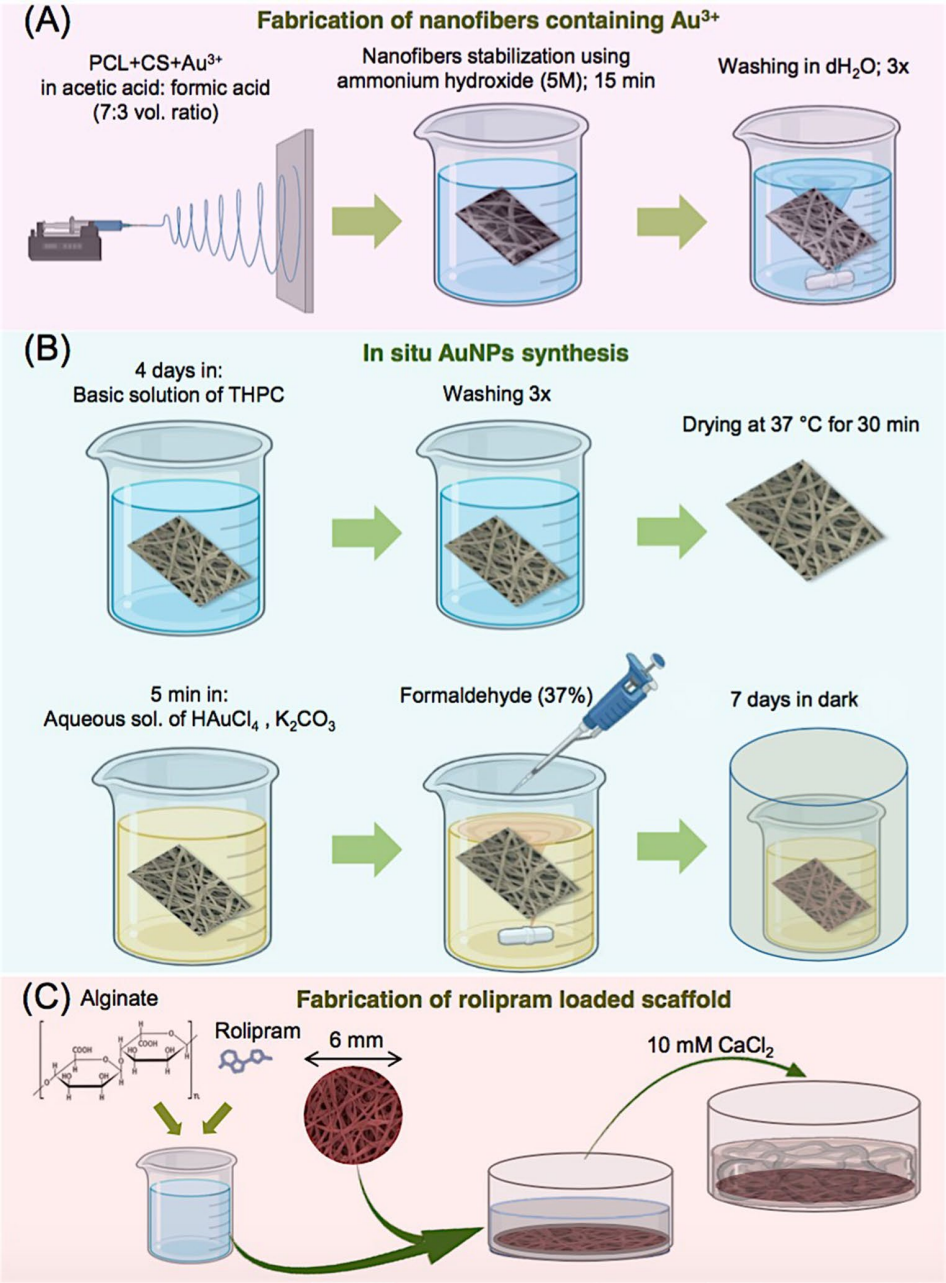
Schematic representation of the steps in conductive 3D scaffold fabrication, including (**A**) fabrication of nanofibrous scaffold based on polycaprolactone (PCL) and chitosan (CS) containing gold ions (Au3+), (**B**) in situ synthesis of gold nanoparticels (AuNPs) within the scaffold, and (**C**) fabrication of rolipram loaded 3D scaffold. THPC: tetrakis (hydroxymethyl) phosphonium chloride

AuNPs were in situ synthesized using two reducing agents: THPC and formaldehyde. The nanofibrous scaffold containing Au ions was immersed in a THPC solution (12 µL THPC (80%), 0.5 mL NaOH (1 M) in 45 mL deionized water) over a period of four days. After three washes and drying at 37 °C for 30 min, the THPC-treated scaffold was immersed in a plating solution of 10 mL comprising 3 mL of HAuCl_4_ (1 wt%) and 200 mL K_2_CO_3_ (1.8 mM), followed immediately by the addition of 50 µL formaldehyde 37% to the mixture. This reaction mixture was kept in a dark place at room temperature for seven days, then rinsed three times and dried at 37 °C for 30 min before use. The schematic presentation of scaffold fabrication is shown in Fig. 1.

The substrates’ biocompatibility was assessed through the MTT assay. Additionally, the nanofibrous scaffold’s morphology and distribution of AuNPs were investigated using field emission scanning electron microscopy (FE-SEM) and energy dispersive X-ray spectroscopy (EDX).

### Cell Metabolic Activity Evaluation in the Rolipram and Electrical Stimulation Groups

Metabolic activity of ADSCs cultured in different conditions was assessed using the MTT assay, as a widely employed test for assessing cell viability, cytotoxicity, and sensitivity to chemotherapy and radiation therapies in in vitro studies [39]. ADSCs were detached using trypsin and then placed in 96-well tissue culture plates at a density of 1 × 10^4^ cells per well. At determined time points, the MTT stock solution (10 µl, 12mM, 5 mg/ml) was added to each well, incubated at 37 °C and 5% CO_2_ for four hours. In order to dissolve the formed formazan crystals, the medium containing MTT reagent was replaced with DMSO (100 µl) and gently shaken for 15 min. Finally, the absorbance (OD) was measured at the wavelength of 570 nm using an ELISA reader. The detailed protocol for the MTT assay is available in the literature [40].

In order to evaluate the effect of rolipram concentration on cell metabolism, ADSCs were seeded in a culture medium containing two concentrations of rolipram (1 µM and 5 µM) for 24 h, as the safe concentrations, which were adapted from a previous report [41]. Based on the results from the previous report [41] and our empirical validation, three concentrations (0.5 µM, 1 µM, and 5 µM) were used for the differentiation process. The electrical density ranging between 50 and 300 mV/mm has been reported as the safe ES regime using different cell types [42–44], and in our case in order to determine the efficienet ES condition ADSCs were stimulated using a frequency of 1 Hz and different voltages, including 50, 100, 200, and 300 mV/mm for two hours/day during 1, 2, and 3 days. MTT assay was conducted 24 h after the last ES.

Additionally, the cells were seeded on conductive scaffolds and exposed to the same ES regimen to evaluate the synergistic effect of scaffold and ES on cell metabolism.

### Rolipram Loading and in Vitro Drug Release Verification

Rolipram was incorporated into the scaffolds using alginate hydrogel. Firstly, a 1% concentration of alginate solution was prepared and filtered through a syringe filter with a 0.22 μm pore size. Different concentrations of Rolipram (0.5, 1, and 5µM) were dissolved in the alginate solution. The scaffolds of 6 mm diameter were sterilized and placed at the bottom of 96-well plates, and then, 50 µl of the alginate solution containing rolipram was poured onto each scaffold. A cross-linking solution of 10 mM CaCl_2_ was added to each well, and the solution was allowed to incubate for approximately 20 min to promote gel formation (Fig. 1C).

For release evaluation, the prepared composite scaffolds were first hydrated by immersion in 1 mL of PBS at 37 °C under gentle shaking for 20 days, and the drug release profile was measured using high-performance liquid chromatography (HPLC), and the calibration curve was prepared using different concentrations of rolipram (5, 7.5, 10, 15, 20, and 25 µM). At predetermined time intervals (i.e., 4, 12 h, and 1, 2, 4, 8, 16 days), 100 µl of samples were withdrawn, and an equal volume of fresh pre-heated PBS was added. The effect of sampling at different time points was applied using the following equation:

$$D_n=C_n+D_{n-1}\left(\frac{V_s}{V_T}\right)$$

where D is the real concentration of the drug in t_n_, C_n_ is the measured concentration in t_n_, D_*n*−1_ is the corrected concentration in t_*n*−1_, V_S_ is the sampling volume (ml), and V_T_ is the receiver medium volume (ml).

### Neuronal Differentiation of ADSCs

Based on the previous section, different concentrations of rolipram (0.5, 1, and 5µM) were added to the cell culture medium and loaded within the conductive nanofibrous scaffolds. ADSCs were seeded at 1 × 10^4^ cells/cm^2^ on rolipram-loaded conductive nanofibrous scaffolds and TCP. The cells were incubated with DMEM/F12 medium supplemented with 10% FBS, 1% penicillin/streptomycin at 37 °C for 24 h. Differentiation of ADSCs was induced by exposing the cells to a pre-induction medium comprised of DMEM/F12 (1:1), 20% FBS, 2% B-27 supplement, 10 ng/ml fibroblast growth factor 2 (FGF2), 250 µM isobutylmethylxanthine (IBME), and 2-mercaptoethanol (100 µM) at 37 °C and 5% CO_2_. After 24 h, the entire cell pre-induction medium was replaced with induction media containing DMEM/F12 (1:1), 0.2% B27, 100 ng/ml of Sonic hedgehog protein (SHH), and 0.01 ng/ml retinoic acid (RA) for 10 days [45]. In the TCP group, rolipram was added to both pre-induction and induction media. The media were changed every three days. The neuronal differentiation was identified by evaluating the expression of β-Tubulin III, a marker for immature neurons, and MAP2, a mature neuronal marker.

### Electrical Stimulation Method

**Fig. 2:**
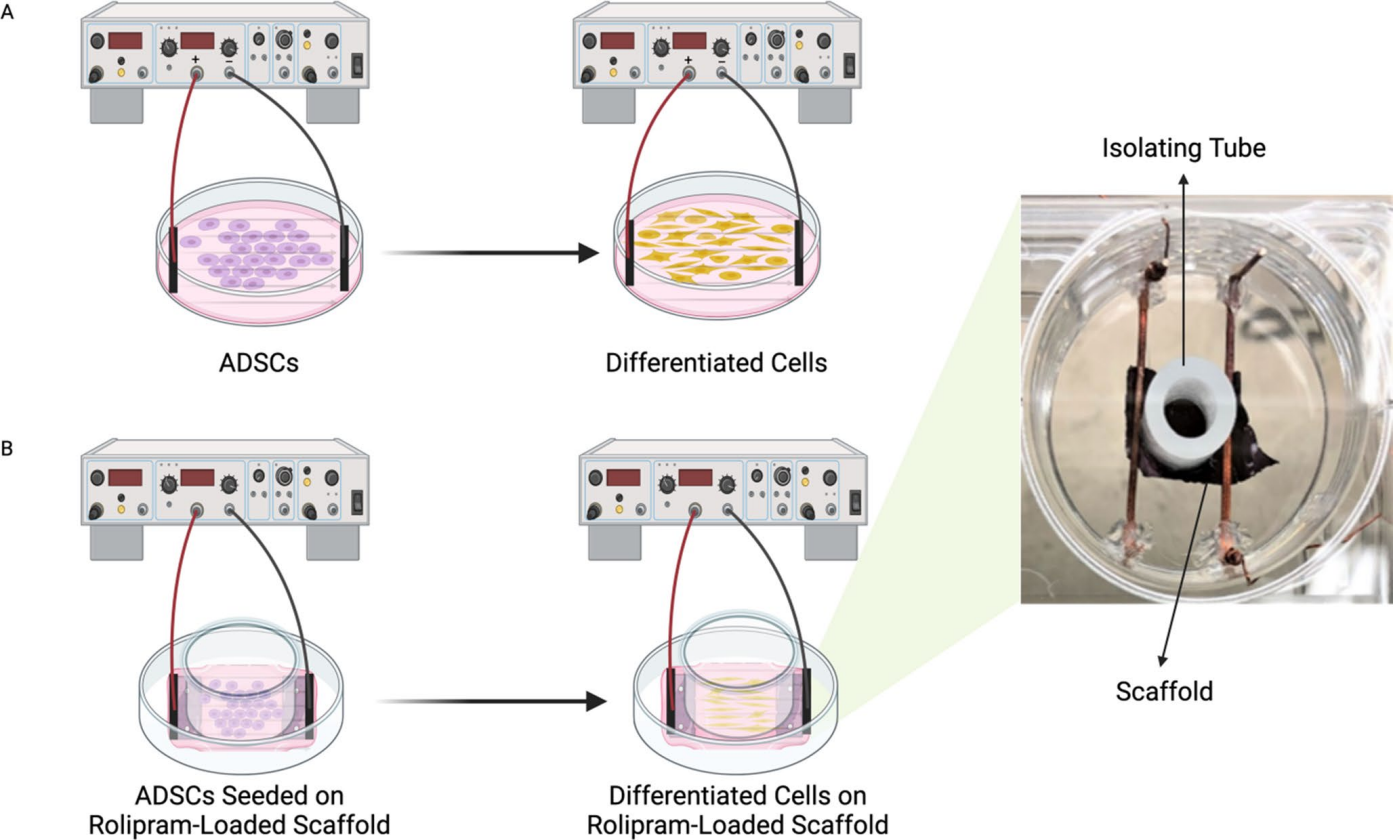
Schematic representation of electrical stimulation setup. Electrical stimulation was applied to the cells cultured in tissue culture plates (**A**) or seeded on rolipram-loaded scaffolds (**B**). ADSC: adipose-derived mesenchymal stromal cell. Figure is created using BioRender.com

Two different setups were used to evaluate the effect of ES on neuronal differentiation of ADSCs: (1) cells were cultured in tissue culture plates, and (2) cells were cultured on the conductive scaffolds (Fig. 2). In the conductive scaffold group, to evaluate the effect of electrical currency via the scaffold instead of the cell culture medium, the electrodes were in direct contact with the scaffold and prevented from being exposed to the cell culture medium, as shown in Fig. 2B. We chose a square wave signal with an electrical density of 100 mV/mm and a frequency of 1 Hz for two hours per day over three days. The selection of an electrical density of 100 mV/mm over three days was based on the results of the metabolic activity of cells exposed to different voltages, which indicated that 100 mV/mm over three days was the most efficient protocol. Two wire electrodes with a circular cross-section of 316 L stainless steel were placed vertically, and two flat wire electrodes with a rectangular cross-section made of copper were placed horizontally and in contact with the scaffold without being in the cultivation environment in each well of the 6-well plates to create the applied voltage. The copper and 316 L stainless steel wires were selected due to their cross-section shape, conductivity, and biocompatibility.

### Immunofluorescence Staining

After the differentiation procedure, the cells were fixed at room temperature using 4% paraformaldehyde in PBS for 30 min and then blocked for 45 min using bovine serum albumin 5% and kept overnight in primary antibody solutions at 4 °C. Each sample was washed three times with PBS/Tween 20. Next, secondary antibodies were added, and samples were kept for one hour in a dark place. Finally, the cells were washed using PBS/Tween 20 (0.1%) and counterstained with DAPI. The primary antibodies were rabbit polyclonal anti-β-Tubulin III (1:500) and anti-MAP2 (1:100 dilution), and the secondary antibody was goat anti-rabbit IgG (H + L) conjugated with FITC (1:2000 dilution). Finally, images were captured using fluorescence microscopy (Olympus BX51, DP71 digital camera). For each experimental group, five random representative areas were selected for neurite length measurement using ImageJ software (https://imagej.net/ij/., National Institutes of Health, Bethesda, MD). The percentage of MAP2 or β-Tubulin III positive cells was determined by dividing the count of marker-positive cells identified by fluorescence intensity above a set threshold by the total count of DAPI-positive cells, then multiplying by 100.

### The Effects of Rolipram and Electrical Stimulation on Intracellular Concentration of cAMP Levels

The ability of rolipram and ES to enhance the intracellular concentration of cAMP was measured using the colorimetric competitive ELISA method following the manufacturer’s instructions for the cAMP kit. A calibration curve was prepared at different cAMP concentrations (1.25, 0.625, 0.312, 0.156, 0.078, 0.039 pmol/50µL) in 0.1 M HCL. Three different groups of cells were evaluated: the cells seeded on a conductive scaffold without rolipram and ES as control, on a conductive scaffold containing 5µM of rolipram, and on a conductive scaffold containing 5µM rolipram and applying ES. Cells seeded on the scaffolds were electrostimulated with a function generator with a square wave signal of 100 mV/mm, 1 Hz frequency, and a duty cycle of 50% for three days, two h/day.

### Statistical Analysis

Data are presented as means ± standard deviations (SD). Independent t-test for comparing two groups. One-way analysis of variance (ANOVA) was used for comparison between more than two groups, and the post hoc assessment was performed using Tukey’s Honest Significant Difference (HSD) to identify pairwise differences between groups. All analyses were conducted using GraphPad Prism 5.04 (GraphPad Software, Inc., CA, USA), and a P-value of < 0.05 was considered statistically significant.

## Results

### Characterization of ADSCs

The morphology of the cells was assessed at the second passage with about 80% confluency. A fibroblast-like morphology was observed (Fig. S1).

The flow cytometry results confirmed the successful isolation of ADSCs from rat adipose tissue (passage 3), which were positive for CD90 and CD105 markers (99.66% and 98.5%, respectively), and negative for CD34 markers (2.28%). The flow cytometry diagram is shown in Fig. S2. Cell morphology was visually assessed via brightfield microscopy. We characterized fibroblast-like morphology by spindle-shaped, elongated cells with bipolar or multipolar extensions.

ADSCs demonstrated successful trilineage differentiation. Oil Red O staining revealed abundant lipid droplets in adipogenically induced cells (Fig. S3). The robust calcium deposition visualized by Alizarin Red S staining confirmed osteogenic differentiation (Fig. S4). Chondrogenic pellets exhibited intense Alcian Blue staining, indicating an extracellular matrix rich in glycosaminoglycans (Fig. S5). These findings confirm the multipotent potential of the isolated ADSCs (Supplementary File 1).

### Characterization of Nanofibrous Conductive Scaffolds

**Fig. 3:**
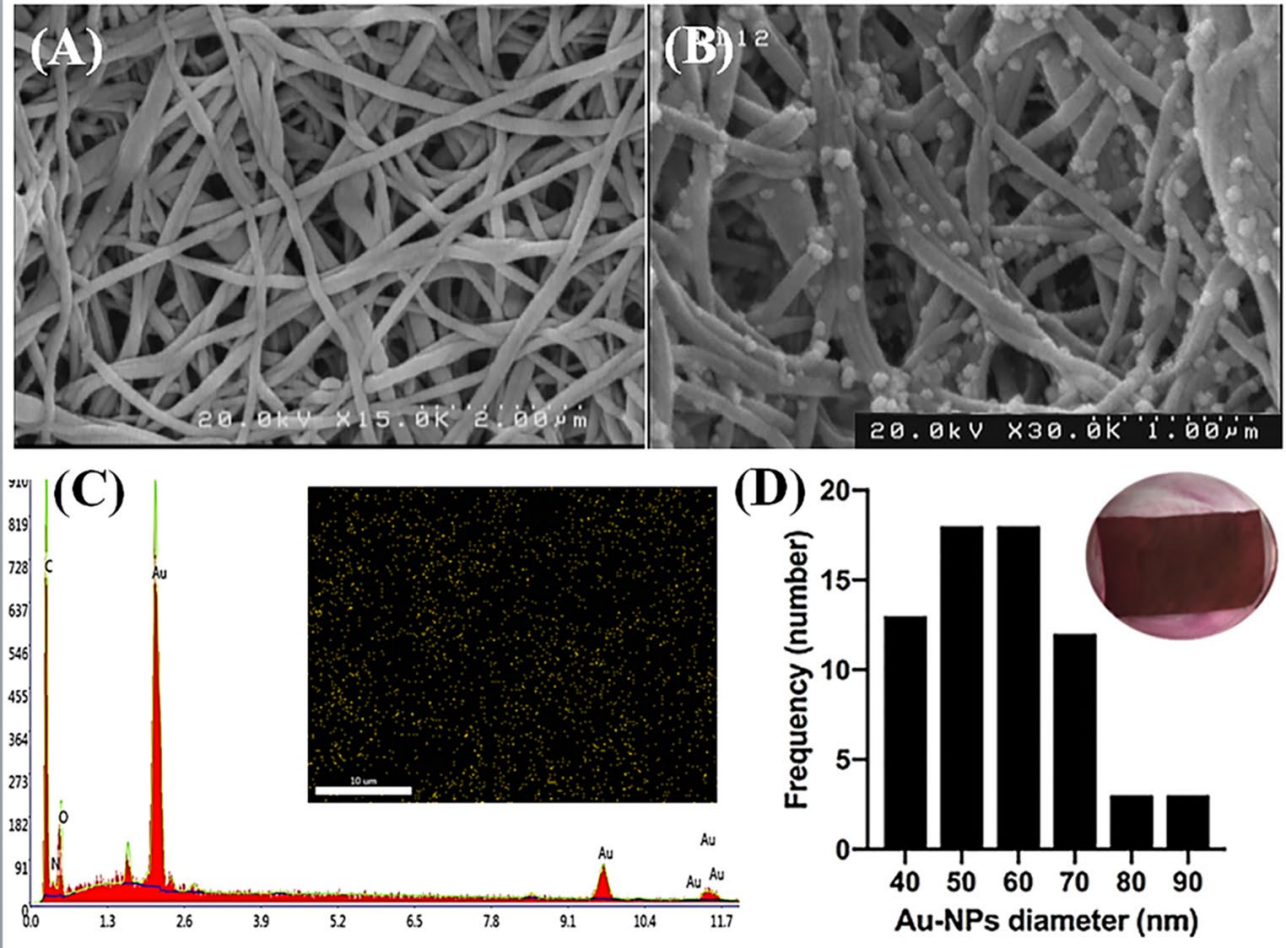
Field emission scanning electron microscopy (FESEM) images of electrospun polycaprolactone (PCL)/chitosan scaffolds: (**A**) morphology after neutralization and (**B**) after gold nanoparticle (AuNP) synthesis. (**C**) X-ray spectroscopy (elemental counts vs. energy in keV) and an inset Au elemental distribution map of PCL/chitosan/AuNP scaffolds, revealing 53.3 wt% Au in the conductive scaffolds. (**D**) Bar plot showing the AuNP size distribution (frequency vs. diameter in nm), with an average AuNP diameter of 57.89 ± 11 nm

The morphology of the scaffold consists of smooth, bead-free fibers with interconnected nanometer-scale pores (Fig. 3A). The AuNPs are firmly embedded in the fiber meshwork without altering the general morphology of the fibers (Fig. 3B). EDX analysis and elemental mapping were performed to provide quantitative and qualitative elemental analyses of the conductive scaffolds and to confirm the presence and distribution of AuNPs on the scaffolds. The elemental mapping identified the location of the Au element in the scaffolds, revealing a uniform distribution of AuNPs throughout the scaffolds (Fig. 3C). The size and distribution of the AuNPs on the scaffold, as well as an optical image of the conductive scaffold have been shown in Fig. 3D. Additionally, the electrical conductivity of the scaffold was measured using the four-point test and found to be 0.12 S.cm^−1^.

### Metabolic Activity of ADSCs Under Different Conditions

**Fig. 4:**
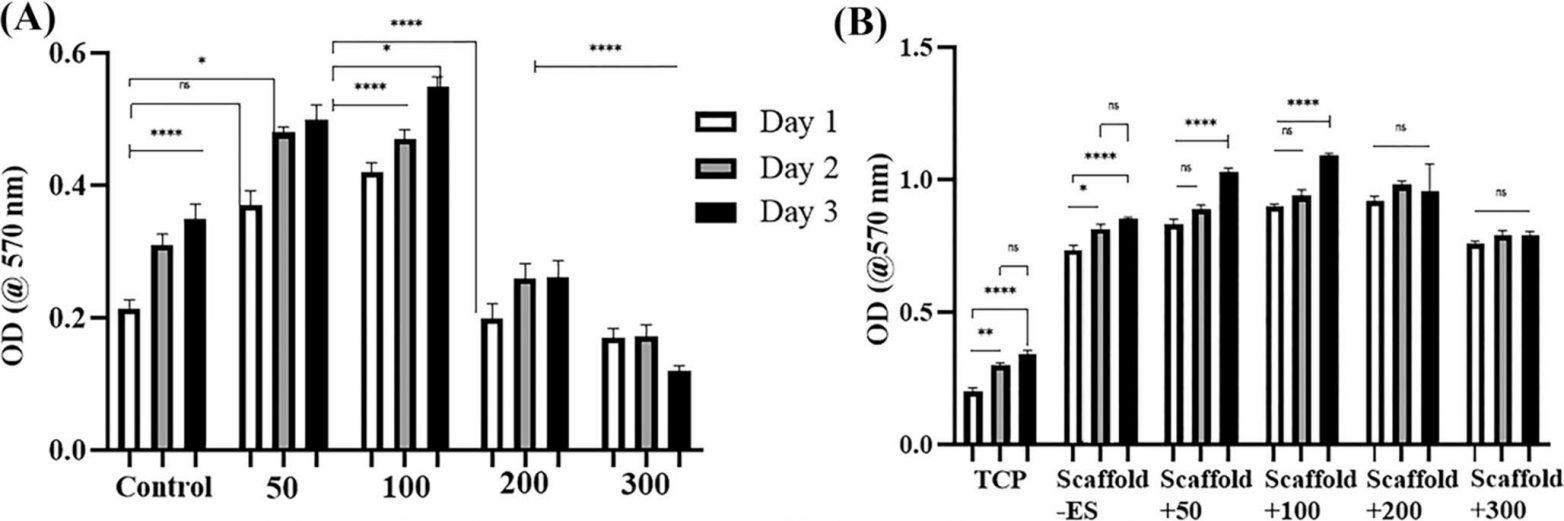
Viability evaluation of cells seeded on tissue culture plate (TCP) or prepared nanofibrous scaffolds under the application of a range of electric field strengths (50–300 mV/mm). Adipose-derived mesenchymal stromal cells (ADSCs) were stimulated for two hours per day over 1, 2, and 3 days, and the MTT assay was conducted 24 h after the final electrical stimulation (ES) session. It shows that the electric densities of 50 and 100 mV/mm are the most efficient for promoting cell proliferation, with a higher electric field resulting in a decrease in optical density (OD) compared to the control group (**A**). When cells were seeded on conductive scaffolds, all groups stimulated with different electric fields showed a significant increase in optical density compared to the TCP. The increase was most prominent in groups exposed to 50 and 100 mV/mm (**B**). (****P < 0.0001, ***P < 0.001, **P < 0.01, *P < 0.05, ns: non-significant, mean ± SD, One-way ANOVA with Tukey’s Post-hoc)

The effect of ES on the metabolic activity of the cells seeded on TCP or conductive scaffolds has been demonstrated in Fig. 4A and B, respectively. The most efficient electrical densities were found to be 50 and 100 mV/mm, while higher voltages (200 and 300 mV/mm) lead to a reduction in optical density compared to the control group at all checked time points (Fig. 4A). Nevertheless, upon seeding cells on conductive scaffolds, a significant increase in optical density was observed in all groups stimulated with different electrical densities and at all time points compared to the TCP. This increase was more prominent in groups exposed to 50 and 100 mV/mm relative to other ones, as shown in Fig. 4B.

**Fig. 5:**
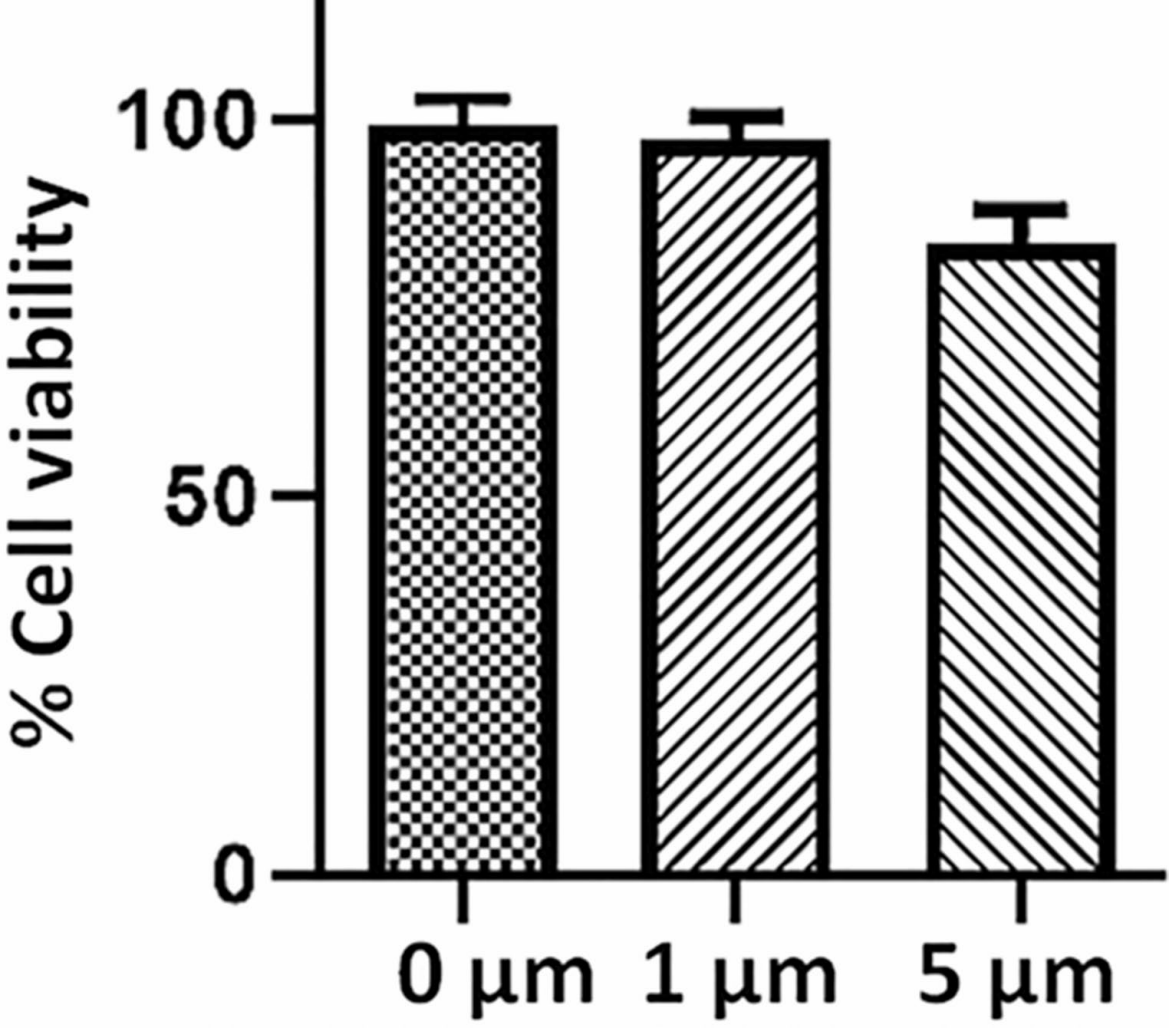
The MTT assay results indicate the impact of two concentrations of rolipram (0, 1 & 5µM) on the viability of ADSCs. After 24 h of exposure, no significant differences in viability were observed among the groups (One-way ANOVA, *p* > 0.05)

Moreover, Fig. 5 shows the effect of different concentrations of rolipram on cell metabolism. Upon exposure of cells to a medium containing 1 and 5 mM rolipram for 24 h, no cytotoxic effect was observed compared to a complete cell culture medium without rolipram.

### In Vitro Drug Release Verification

**Fig. 6:**
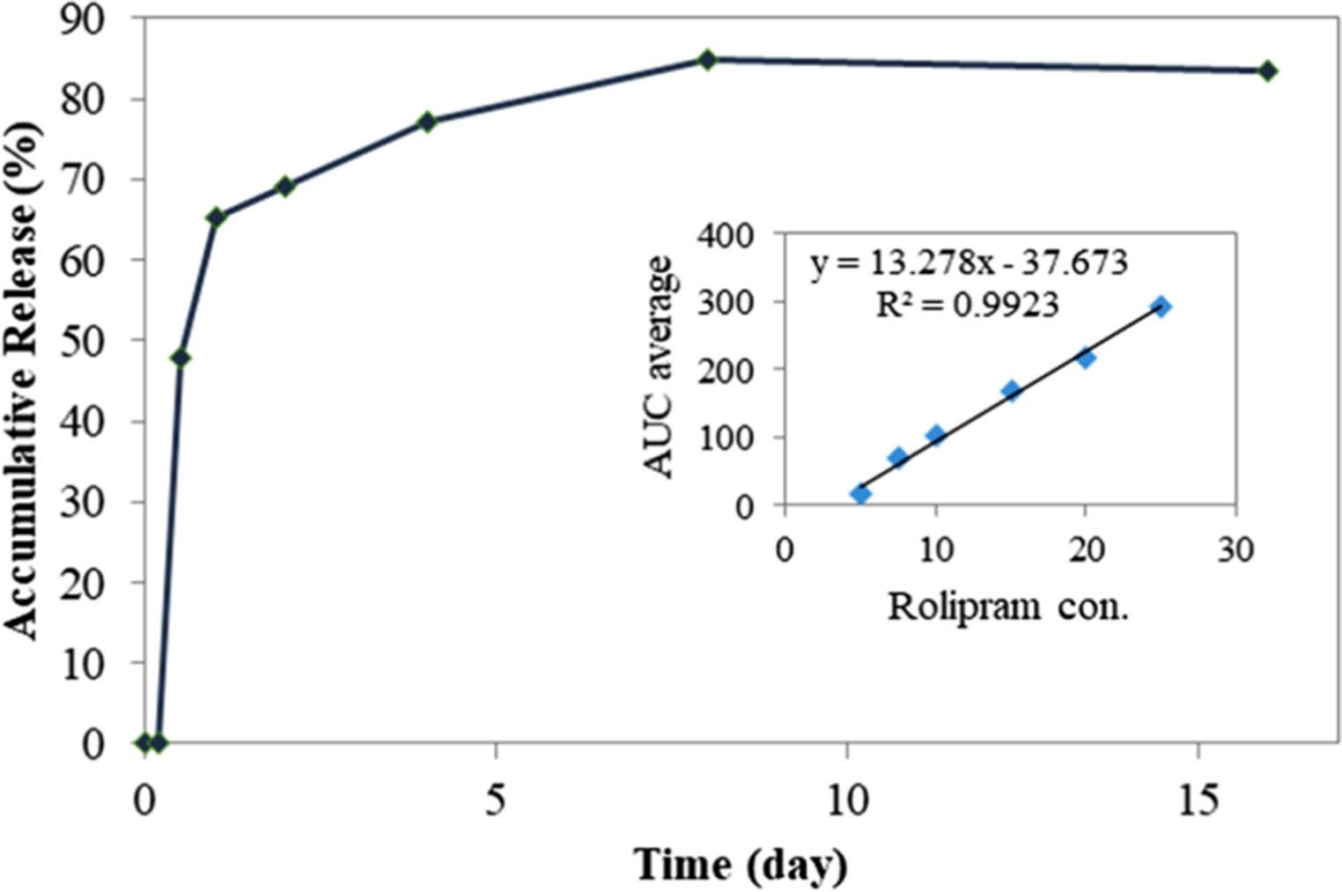
Rolipram release profile from nanofibrous scaffold. The nanofibrous scaffold demonstrated an initial burst release of rolipram on the first day, followed by sustained release until day 8. This release profile is beneficial for the 10-day differentiation protocol and effectively controls the release of rolipram using alginate hydrogel. The insert shows the calibration curve. AUC: area under the curve

The release profile of rolipram from the scaffold was analyzed using HPLC (Fig. 6). A range of rolipram concentrations (5, 7.5, 10, 15, 20, and 25 µM) was employed to generate the calibration curve. The release profile displayed an immediate surge in release on day one, with sustained release continuing until day 8. This release profile is advantageous for the 10-day differentiation protocol, demonstrating efficient control of rolipram release using alginate hydrogel.

### Neuronal Differentiation of ADSCs Using Different Concentrations of Rolipram

**Fig. 7:**
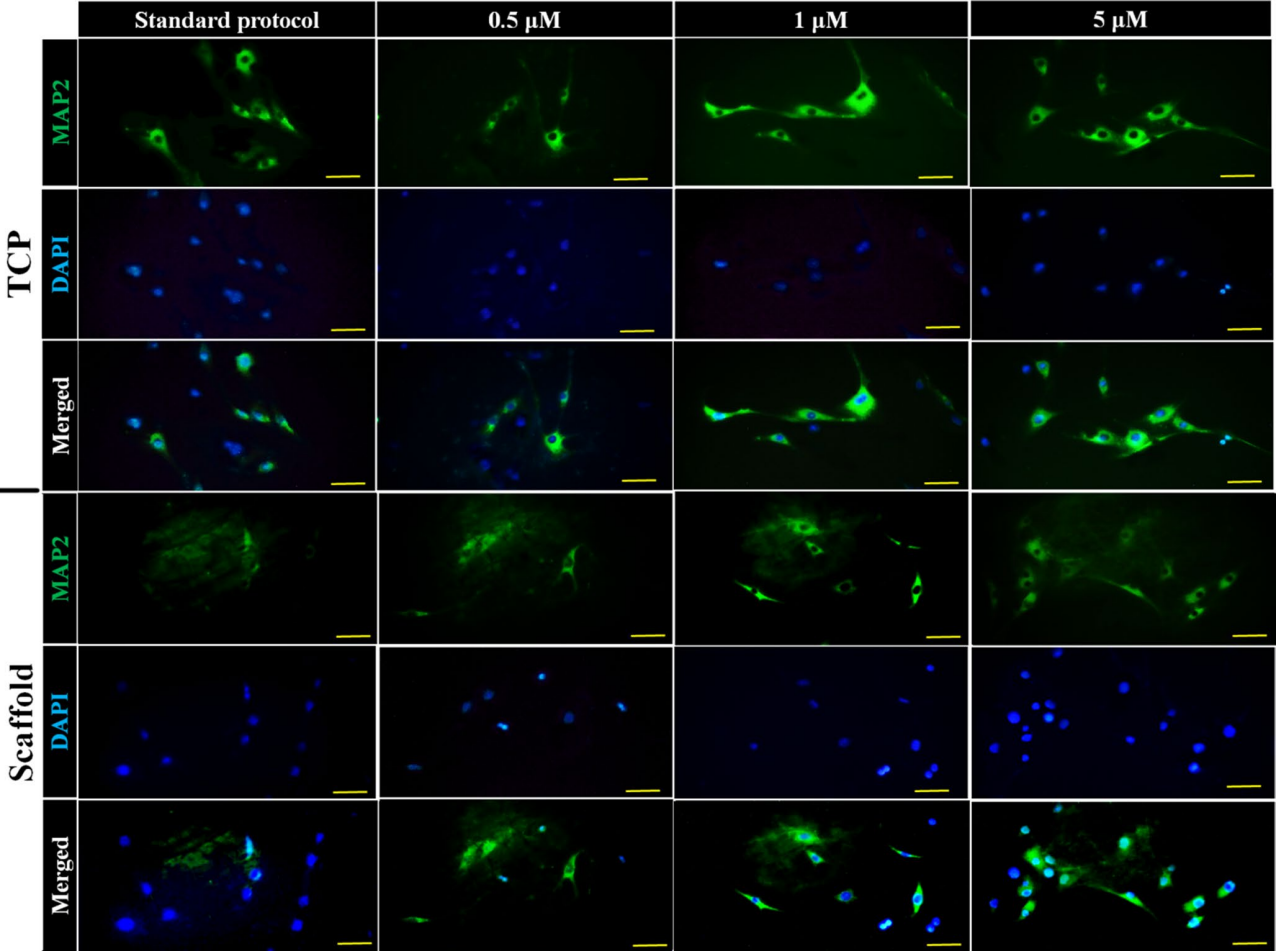
Fluorescent microscopy illustration of MAP2 (neuronal marker) shows the effect of different concentrations of rolipram (0.5, 1, 5 µM) on the neuronal differentiation of adipose-derived mesenchymal stromal cells (ADSCs) seeded on tissue culture plates (TCP) or rolipram-loaded conductive nanofibrous scaffolds (Scaffold) in comparison to standard protocol of neuronal differentiation using sonic hedgehog protein (SHH) and retinoic acid (RA) without rolipram. Scale bar equals 20 μm

**Fig. 8:**
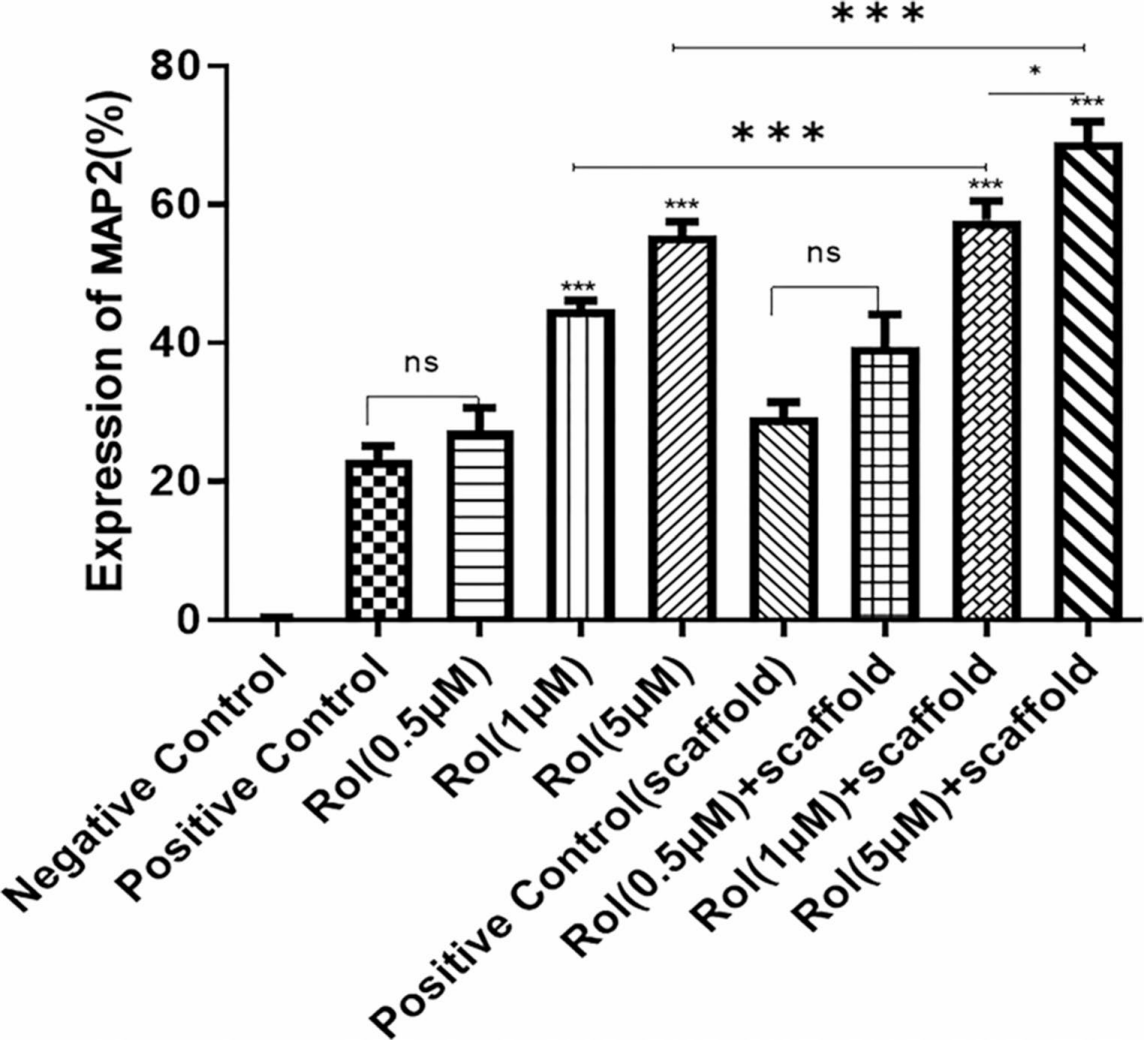
Quantitative evaluation of MAP2-positive cells at different concentrations of rolipram (Rol) with and without scaffold. (***P < 0.001, *P < 0.05, ns: non-significant, mean ± SD, One-way ANOVA with Tukey’s Post-hoc test)

The inclusion of 0.5 µM rolipram in the cell culture medium did not cause a significant change in the percentage of MAP2 and β-Tubulin III positive cells compared to the control group, which utilized the standard protocol of neuronal differentiation involving SHH and RA. However, when the rolipram concentration was increased to 1 µM, a statistically significant increase in the percentage of cells positive for MAP2 (2.4-fold) and β-Tubulin III (3.4-fold) was observed. The percentage of MAP2-positive cells showed a 2.4-fold increase, and the percentage of β-Tubulin III-positive cells showed a 4-fold increase in cells treated with 5 µM rolipram, indicating an even more pronounced increase. Furthermore, the immunofluorescent staining of MAP2 and β-Tubulin III in cells seeded on scaffolds containing rolipram demonstrated that drug encapsulation within the scaffold and sustained release of rolipram could enhance neuronal differentiation, particularly at higher concentrations. The impact of varying concentrations of rolipram on the expression of β-Tubulin III, a marker for immature neurons, and MAP2, a mature neuronal marker, in cells cultured on TCP and conductive scaffolds was shown in Fig. 7, and its quantification is reported in Fig. 8.

A substantial difference was revealed in ADSC differentiation at 1 µM and 5 µM drug concentrations compared to the control (i.e., using standard differentiation approach) (*P* < 0.001) in both groups (with and without scaffold). Additionally, the number of differentiated ADSCs in the 5 µM scaffold group was significantly greater (*P* < 0.05) than in the 1 µM group, although this difference was not statistically significant in the non-scaffold group. Moreover, the difference between 0.5 µM and the control in both groups was not statistically significant. Between-group analysis also demonstrated a statistically significant difference (*P* < 0.001) in the 1 µM and 5 µM concentrations of the scaffold group compared to the non-scaffold group (Fig. 7). Therefore, the optimal concentration of 5 µM was determined to be suitable for further investigation with ES.

**Fig. 9:**
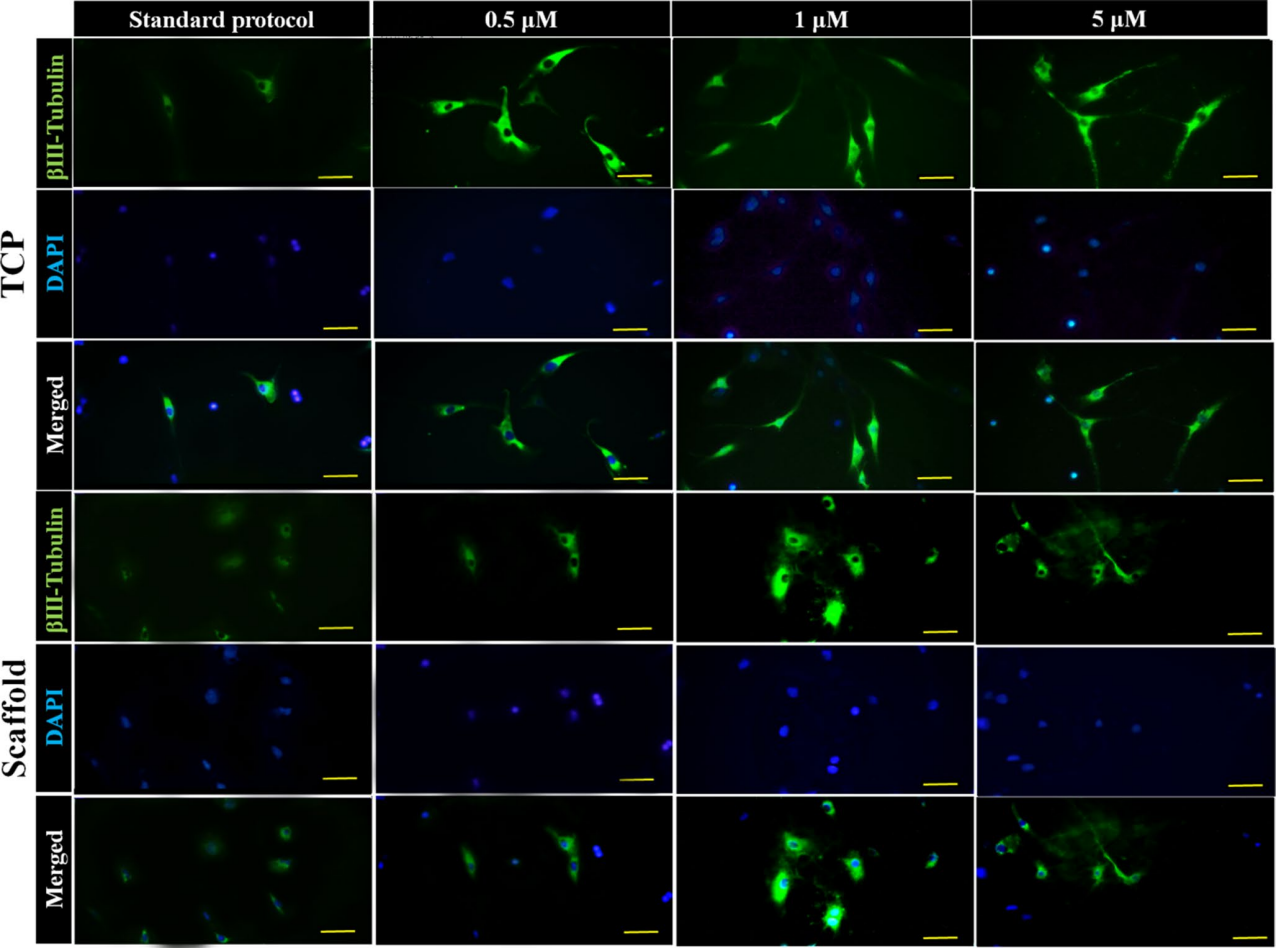
Fluorescent microscopy illustration of β-Tubulin III marker shows the effect of different concentrations of rolipram (0.5, 1, 5 µM) on the neuronal differentiation of ADSCs seeded on tissue culture plates (TCP) or rolipram-loaded conductive nanofibrous scaffolds (Scaffold) in comparison to standard protocol of neuronal differentiation using SHH and RA without rolipram. Scale bar equals 20 μm

**Fig. 10:**
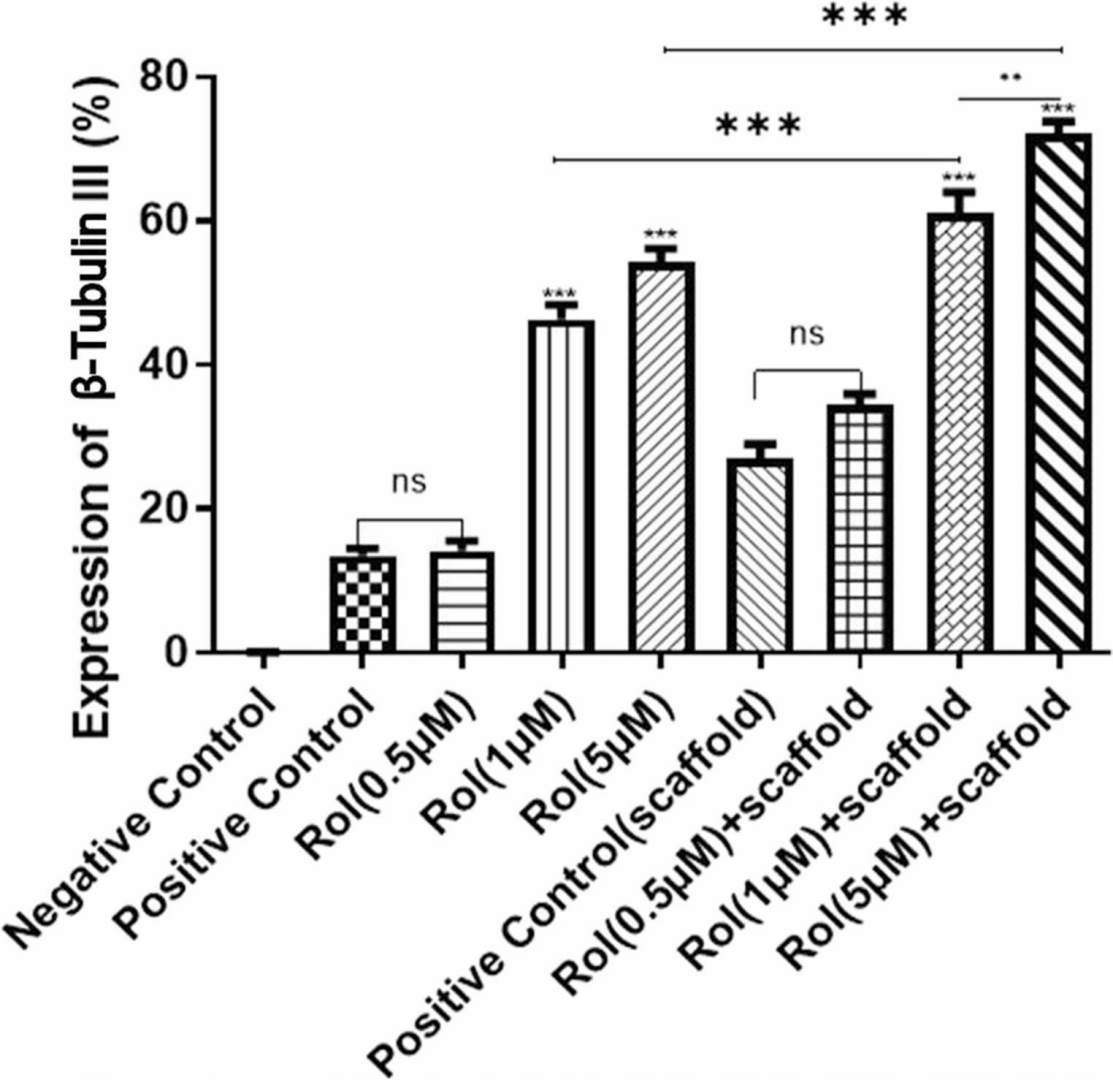
Quantitative evaluation of β-Tubulin III positive cells at different concentrations of rolipram (Rol) with and without scaffold. (***P < 0.001, *P < 0.01, ns: non-significant, mean ± SD, One-way ANOVA with Tukey’s Post-hoc test)

Immunocytochemical staining for β-Tubulin III was also performed and analyzed similarly to the MAP2 marker, and comparable results were obtained for the same concentrations of rolipram in the two groups of differentiation compared to the control (Fig. 9). The expression of β-Tubulin III in different groups is quantitatively compared in Fig. 10.

### Synergistic Effect of Electrical Stimulation and Rolipram on Neuronal Differentiation of ADSCs on the Conductive Nanofibrous Scaffold

**Fig. 11:**
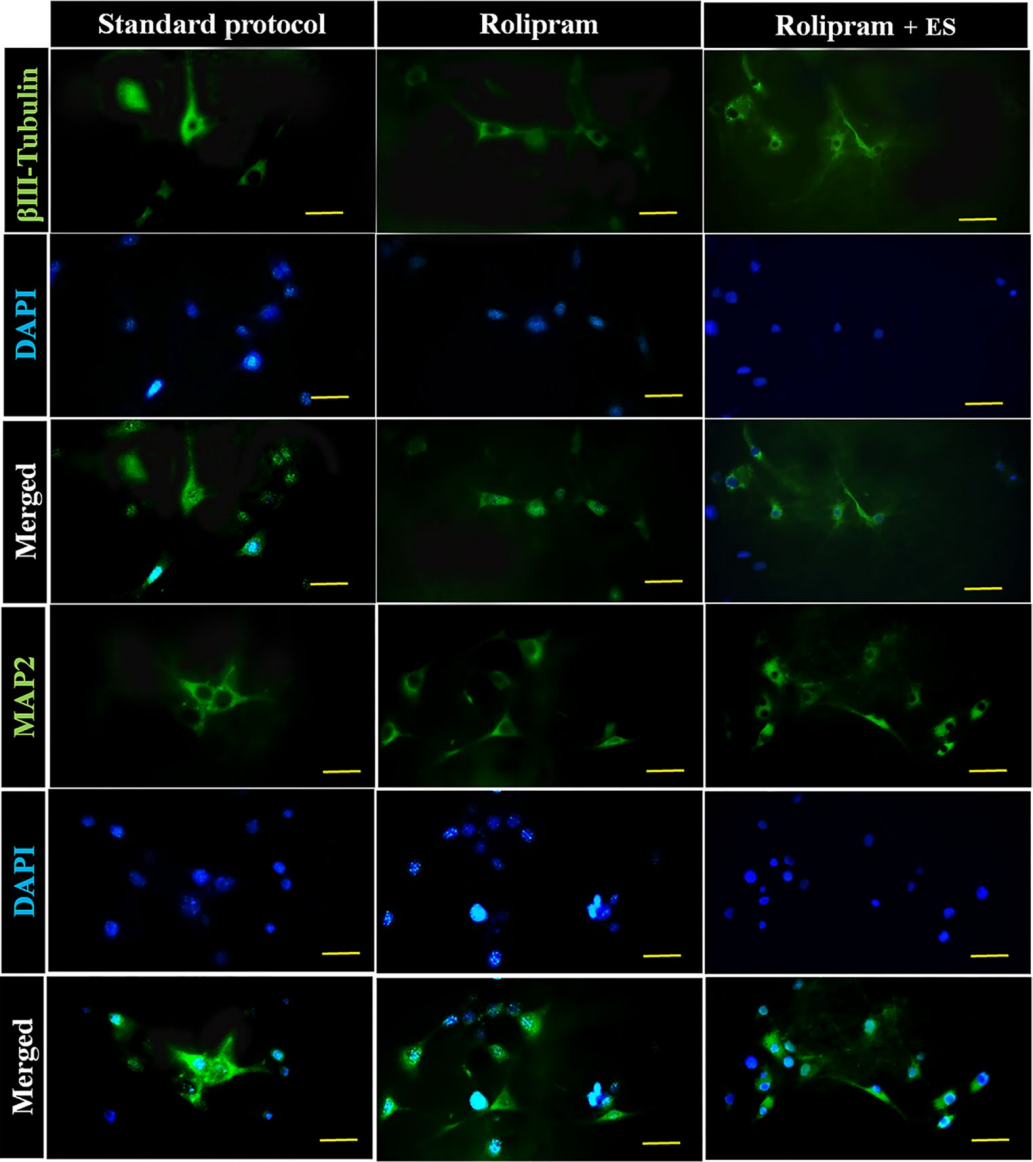
Fluorescent microscopy of cells seeded on conductive scaffold and subjected to neuronal differentiation using a standard protocol with the addition of rolipram and electrical stimulation to detect the presence of positive cells for β-Tubulin III and MAP2

To assess the synergistic effect of sustained release of rolipram from a conductive nanofibrous scaffold and ES on the neuronal differentiation of ADSCs, the expression of MAP2 and β-Tubulin III markers was measured in three experimental groups: the control group (cells differentiated using the standard protocol), cells differentiation on a rolipram-loaded scaffold, and cells differentiation on a rolipram-loaded scaffold under ES. The optimal concentration of 5 µM rolipram was used for electrical stimulation of ADSCs at 100 mV/mm (Fig. 11).

**Fig. 12:**
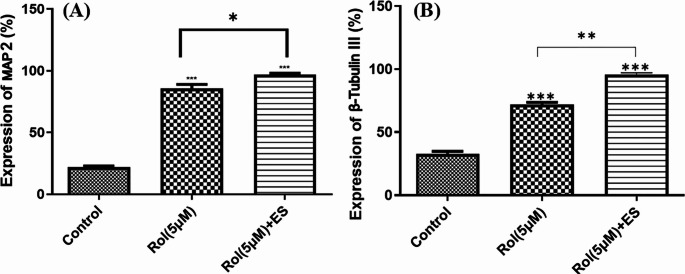
MAP2 (**A**) and β-Tubulin III (**B**) expressions in ADSCs seeded on the conductive nanofibrous scaffold after differentiation using standard protocol alone or in combination with rolipram (5µM) and rolipram (Rol) plus ES (electrical stimulation) (100 mV/mm) (***P < 0.001 compared to the control group, **P < 0.01 and *P < 0.05 compared to 5µM rolipram group, mean ± SD, One-way ANOVA and with Tukey’s Post-hoc test)

The statistical analysis revealed a significant increase (*P* < 0.001) in the expression of the neuronal cell markers MAP2 and β-Tubulin III in both the rolipram and rolipram plus ES groups, as compared to the control group. Furthermore, analysis of the electrically stimulated group indicated a significant increase in the ADSCs differentiation potential (*P* < 0.05) induced by the 5 µM rolipram and ES scaffolds compared to the rolipram alone group (Fig. 12A & B).

### cAMP Level Confirms the Induction Potential of Rolipram and Electrical Stimulation

**Fig. 13:**
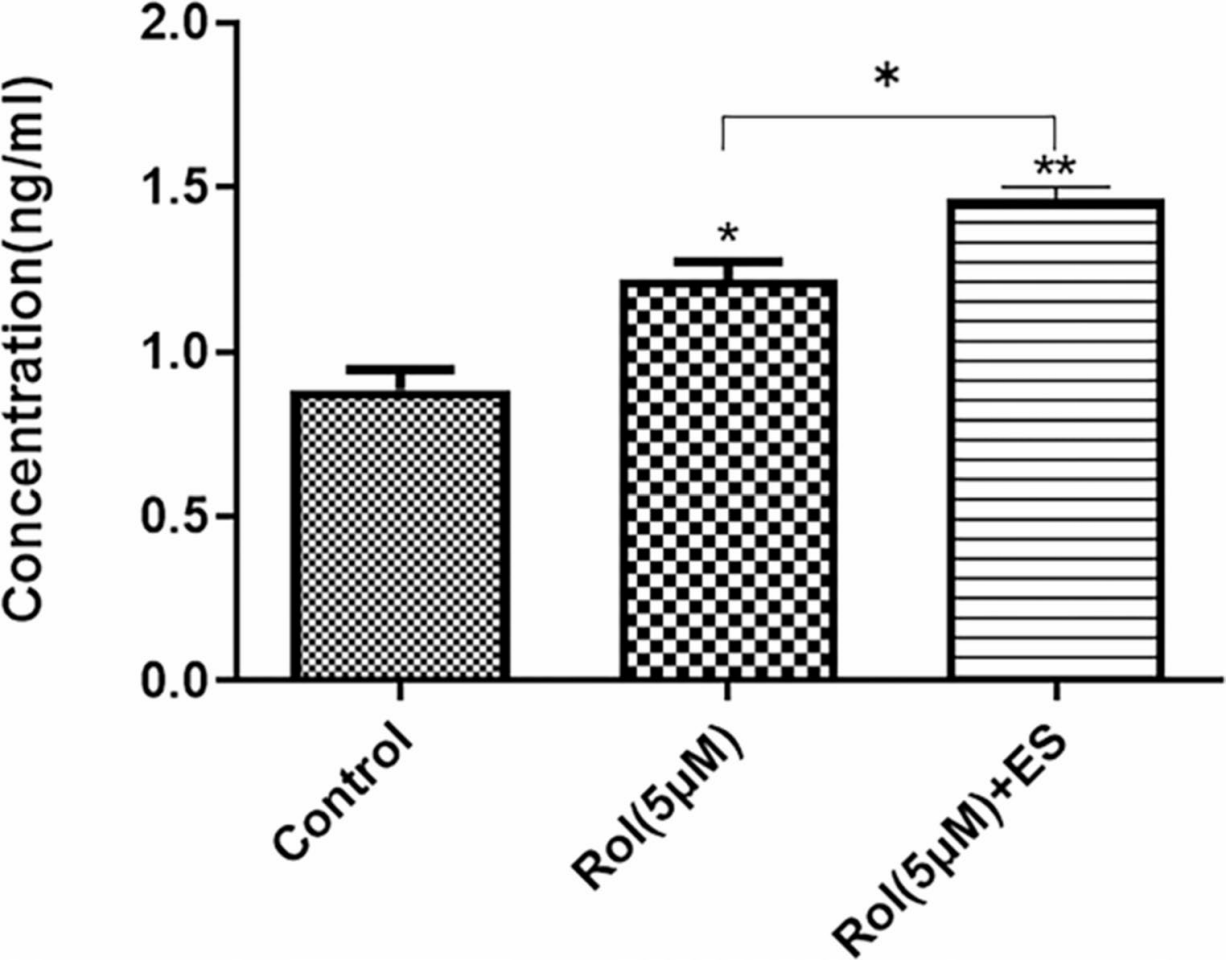
Measurement of cAMP production by ADSCs under different conditions. The three groups include the control without nanofibrous scaffolds, 5µM rolipram-treated nanofibrous scaffolds, and 5µM rolipram (Rol) + electrical stimulation (ES) treated nanofibrous scaffolds. cAMP levels were significantly higher in 5µM rolipram and 5µM rolipram + ES groups (One-way ANOVA with Tukey’s Post-hoc test, *P < 0.05, **P < 0.01, mean ± SD)

The measurement of cAMP levels in ng/mL demonstrated a significantly elevated concentration in the cells differentiated under different conditions including without nanofibrous scaffolds as control, 5µM rolipram-treated nanofibrous scaffolds, and 5µM rolipram + ES treated nanofibrous scaffolds (Fig. 13). The results showed that ES caused a further increase in cAMP levels compared to the 5 µM rolipram-only group (*P* < 0.05).

## Discussion

The limited regenerative ability of neural tissue has made its treatment challenging; cell therapy and tissue engineering have emerged as effective therapeutic options. While cell therapy and tissue engineering offer promising avenues for repair, developing scaffolds that effectively mimic the native nerve tissue’s ECM, release therapeutic agents, and provide facilitative cues like electrical conductivity is crucial. In the present study, we aimed to develop a conductive nanofibrous PCL/chitosan/Au-NP scaffold loaded with rolipram, a phosphodiesterase IV (PDE4) inhibitor. Our primary goal was to investigate the synergistic effect of sustained rolipram release and ES on the in vitro proliferation and neural differentiation of ADSCs, a readily accessible and abundant cell source for therapeutic applications. To achieve this, we first optimized the rolipram concentration and Electrical field density to ensure minimal cytotoxicity while maximizing the potential for neural differentiation. Alginate hydrogel was used to load and sustain the release of rolipram. While existing evidence suggests rolipram and ES can enhance axon elongation and nerve regeneration in vivo [46, 47], our investigation focused specifically on their combined impact on the in vitro neuronal differentiation process of ADSCs rather than on their regenerative potential.

The selection of appropriate rolipram dosage and ES parameters was critical. Downing et al. showed that loading lower doses of rolipram (3.1–3.9 µg/cm^2^) in nanofibrous scaffolds results in enhanced nerve regeneration and functional recovery compared to higher concentrations [48]. In our in vitro study, no cytotoxic effect on ADSCs was found by using rolipram concentrations up to 5 µM, and this concentration was used for scaffold loading and neural differentiation assessments. An electrical density of 100 mV/mm was also found to be safe and efficient for neural differentiation, in line with previous reports [49, 50].

A key finding of our study was that the fabricated conductive nanofibrous scaffold itself supported ADSC proliferation, and this pro-proliferative effect was further augmented by the sustained release of rolipram and the application of ES. We hypothesized that rolipram and ES would synergistically elevate intracellular cyclic adenosine monophosphate (cAMP) levels, in line with other studies [46, 47]. Consistent with this hypothesis, our quantitative assessments revealed that the concurrent administration of 5µM rolipram and ES (100 mV/mm) via the conductive scaffolds led to a substantially higher cAMP concentration when compared to treatments involving either rolipram alone or the control setup. This biochemical enhancement was mirrored in the expression of neuronal markers; the combination treatment led to the highest percentage of MAP2 and β-tubulin III-positive cells, indicating a more robust neuronal differentiation.

While ES has demonstrated potential for improving the regeneration of the peripheral nervous system, its use in regenerating the central nervous system is still in the early stages. Studies have shown that in vivo ES for one hour after nerve injury results in upregulation of genes such as growth-associated protein-43 (GAP-43) and BDNF [28, 30, 51]. This process is continued by elevated intracellular cAMP levels in stimulated neurons. The initial increase in cAMP levels is likely caused by increased calcium entry into the neuronal cell body [52]. Previous research by Garrudo et al. (2021) demonstrated that ES enhanced neural maturation on PCL/polyaniline scaffolds with a conductivity of 0.063 ± 0.029 S.cm^−1^ [49]. In the present study, our PCL/chitosan/Au-NP nanofibrous scaffolds achieved a higher conductivity of 0.12 S.cm^−1^ and showed no cytotoxic effects on ADSCs. This improved conductivity, combined with biocompatibility, suggests our scaffold may offer enhanced potential for tissue engineering applications compared to previously reported materials.

Dong et al. demonstrated that long-term ES caused a significant increase in the proportion of neuronal differentiation of rat filum terminal-derived neural progenitor cells (FT-NPCs) in vitro. In their study, the cells received a 12-hour stimulation on the first day, then a daily 2-hour treatment with ES until the end of the experiment in a cell culture medium containing DMEM/F12 + 10% FBS. Also, they revealed that the use of 150 mV/mm significantly increased their neuronal differentiation rate in rats [53]. Addition of growth factors to the cell culture medium during the ES can shorten the time to reach an efficient neural differentiation, and in a study, 10 min/day ES of NSCs along with EGF/FGF or IFN-c resulted in longer neurites, mature neuronal morphologies, and signs of differentiation compared to those without stimulation [54]. Another in vitro study [55] showed that a combination of ES and copper in the cell culture medium can induce stromal cell differentiation towards the neuronal lineage, resulting in elongated cells and upregulation of neuron-specific genes and proteins. However, in their study [55], cell viability decreased to 78 ± 11% after the one-hour stimulation. The highest expression of β-Tubulin III was observed on day seven when cells were stimulated with 1.5 mA in a cell culture medium containing Cu. Positive expression of both β-Tubulin III and MAP-2 was observed only when ADSCs were stimulated with a combination of copper and electrical current [55].

**Fig. 14:**
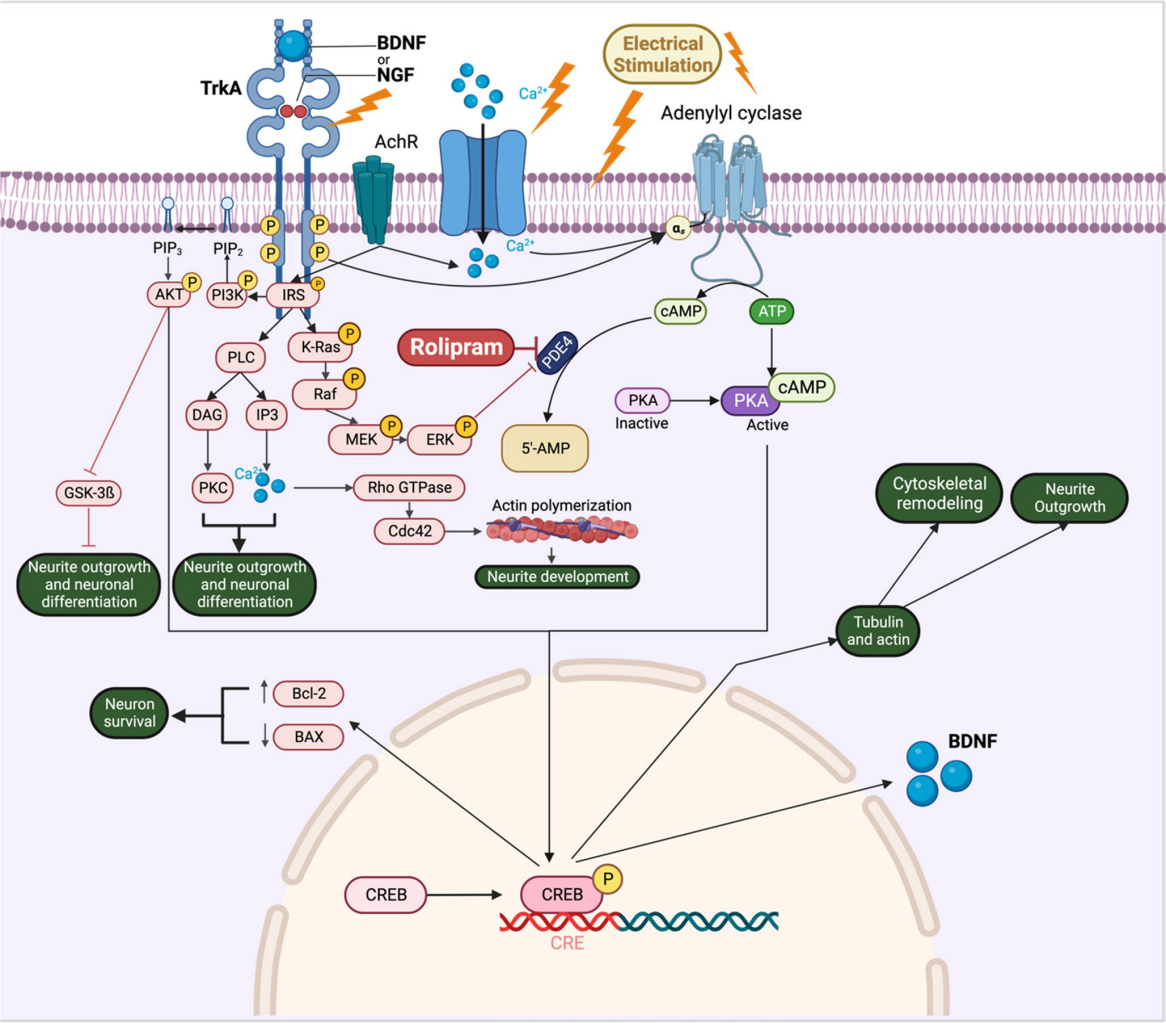
Summary of pathways through which ES and rolipram induce neuronal differentiation. ES could activate the cAMP pathway by stimulating adenylyl cyclase. In addition, rolipram could prevent degradation of the cAMP by inhibiting the PDE4. The activated cAMP pathway leads to increased expression of cytoskeleton proteins, neurite outgrowth, and nerve regeneration. Also, the ES improves Ca^2+^ influx, which can improve neuronal differentiation. Additionally, the cAMP pathway upregulates BDNF expression, enhancing cell viability and promoting neuronal differentiation. The Figure is created using BioRender.com. IRS: Insulin Receptor Substrate; PIP2: Phosphatidylinositol 4,5-bisphosphate; PIP3: Phosphatidylinositol [3–5]-trisphosphate; Ca²⁺: Calcium ion; ATP: Adenosine Triphosphate; cAMP: Cyclic Adenosine Monophosphate; AKT: Protein Kinase B; PI3K: Phosphoinositide 3-Kinase; PLC: Phospholipase C; DAG: Diacylglycerol; IP3: Inositol 1,4,5-trisphosphate; K-Ras: Kirsten Rat Sarcoma Viral Oncogene Homolog; Raf: Rapidly Accelerated Fibrosarcoma kinase; MEK: Mitogen-Activated Protein Kinase Kinase; ERK: Extracellular Signal-Regulated Kinase; PKC: Protein Kinase C; PKA: Protein Kinase A; PDE4: Phosphodiesterase 4; Rho GTPase: Rho family of small GTP-binding proteins; Cdc42: Cell Division Control Protein 42 homolog; GSK-3β: Glycogen Synthase Kinase 3 Beta; CREB: cAMP Response Element-Binding Protein; BAX: Bcl-2 Associated X Protein; Bcl-2: B-cell lymphoma 2; BDNF: Brain-Derived Neurotrophic Factor; 5’-AMP: 5’-Adenosine Monophosphate; CRE: cAMP Response Element

In the current study, we studied the synergistic effect of ES and rolipram on a previously developed protocol [23, 45] for neural differentiation and showed that adding these physical and chemical cues can facilitate neural differentiation of ADSCs and elevate the levels of cAMP. ES has been shown to function via a pathway akin to the retrograde intracellular Ca^2+^ current that arises after axotomy [56, 57]. Also, ES can improve Ca^2+^ influx and subsequently activate adenylyl cyclase. It is suggested that ES could increase the production of BDNF and nerve growth factor (NGF) and their receptor, tropomyosin receptor kinase B (trkB), which can have various roles in normal neural development and plasticity [58–60]. Rolipram inhibits the PDE4 and augments cAMP pathways. BDNF could also inhibit PDEs through mitogen-activated protein kinases (MAPK)/extracellular signal-regulated kinases (ERK) pathway, hence preventing degradation of the cAMP [61, 62]. BDNF boosts the cAMP pathway through a Ca^2+^-dependent mechanism, enhancing the expression of proteins, including β-Tubulin III and GAP-43. Cytoskeletal assembly and remodeling are improved by activating the cAMP response element binding (CREB) [62, 63]. A summary of ES and rolipram mechanisms in neuronal differentiation has been shown in Fig. 14.

Despite promising results, this study has limitations. As an in vitro investigation, it may not fully replicate in vivo conditions, highlighting the need for animal studies. Although cAMP levels and neuronal markers were assessed, detailed molecular analyses, such as Polymerase Chain Reaction (PCR) or Western blotting, need to be performed. Long-term effects of ES and scaffold degradation were also not evaluated. Additionally, issues related to scaffold scalability, reproducibility, and regulatory approval were beyond the study’s scope and warrant future investigation.

## Conclusions

Developing effective therapies for traumatic spinal cord injuries is challenging due to the complexity of nerve tissue and limited regenerative abilities. Our findings demonstrate that the fabricated PCL/chitosan/Au-NP conductive scaffold, when loaded with rolipram and subjected to ES, significantly enhanced the in vitro neural differentiation of ADSCs. Specifically, this combinatorial approach led to an increased percentage of cells expressing the neuronal markers MAP2 and β-tubulin III, and notably, was associated with elevated cAMP levels. These results indicate that the synergistic application of rolipram and ES on a conductive biomaterial platform can effectively facilitate the differentiation of ADSCs towards a neuronal lineage. This combinatorial strategy presents a promising in vitro model that could contribute to the development of novel therapeutic approaches for nerve regeneration, particularly in the context of traumatic spinal cord injuries. However, additional research is recommended to confirm these findings in preclinical studies, and future investigations should assess in vivo efficacy, scaffold longevity, and underlying molecular pathways to support clinical translation.

## Electronic Supplementary Material

Below is the link to the electronic supplementary material.

Supplementary Material 1

## Data Availability

The datasets used and/or analyzed during the current study are available from the corresponding author upon reasonable request.
