# Supplementary material for: Rolipram and Electrical Stimulation Synergistically Promote Neuronal Differentiation of Adipose-derived Stromal Cells: an in Vitro Study
Source: Stem Cell Rev Rep. 2025 Jun 26;21(7):2218–36. doi: 10.1007/s12015-025-10925-5 (PMC12408716; doi:10.1007/s12015-025-10925-5)
Supplement: Supplementary file 1 — Supplementary Material 1 [file 12015_2025_10925_MOESM1_ESM.docx]

**Adipose Mesenchymal Stromal Cells Characterization**

**Fibroblast-Like Morphology:**

Following the second passage, the cells were cultured. Once they reached approximately 80% confluency, they were examined under a microscope, revealing a fibroblast-like morphology.

**
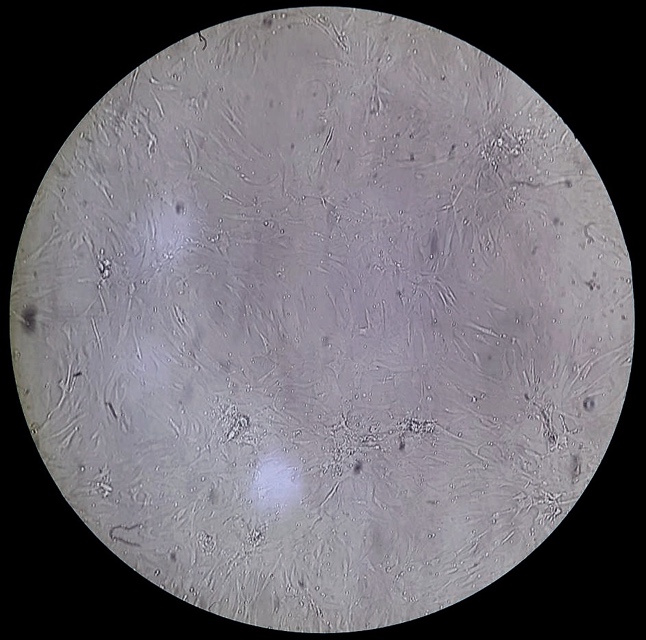
**

**Fig. S1.** Morphology of fibroblast-like adipose mesenchymal stromal cells.

**Flowcytometry Evaluation:**

The flow cytometry results confirmed the successful isolation of ADSCs from rat adipose tissue (passage 3), which were positive for CD90 and CD105 markers (99.66% and 98.5%, respectively), and negative for CD34 markers (2.28%). The flow cytometry diagram is shown in Fig. S2.


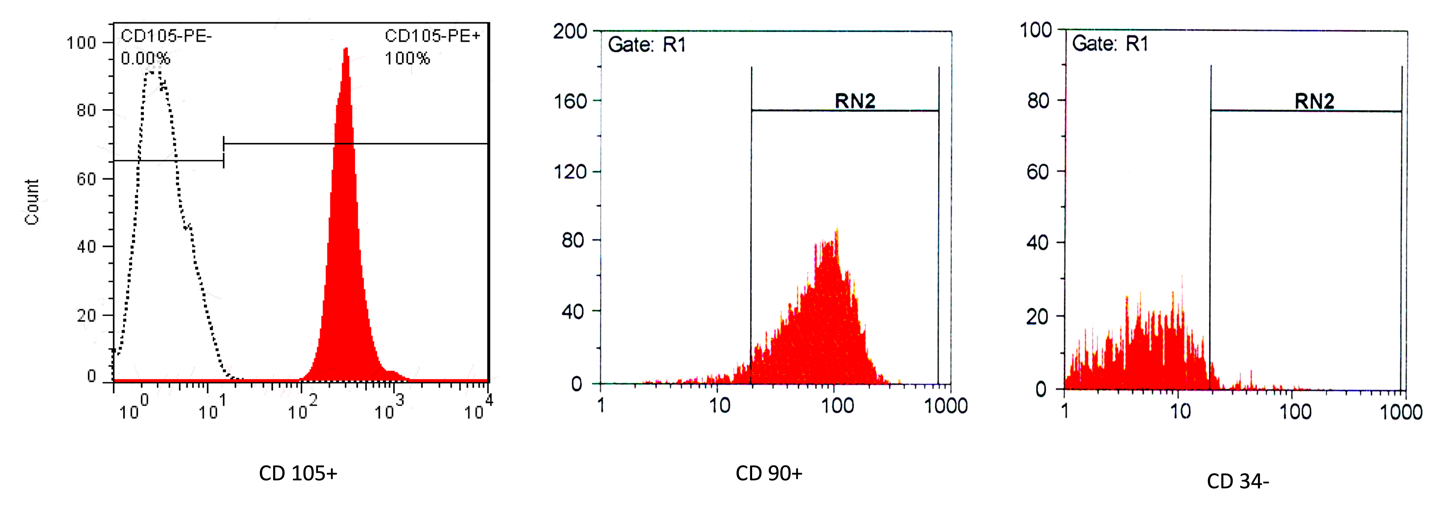
**Fig. S2. Characterization of adipose-derived stromal cells (ADSCs) by flow cytometry.** Representative histograms show high expression of mesenchymal stem cell markers CD105 and CD90, and low/negative expression of hematopoietic marker CD34, confirming the mesenchymal lineage of the isolated ADSCs.

**Adipogenic Differentiation:**

When the cells reached 80% confluency, they were induced for adipogenic differentiation using Ham’s/F12 medium supplemented with 10% FBS, 250 nM dexamethasone, 0.5 mM Isobutylmethylxanthine, and 0.2 mM indomethacin. After three weeks, the cells were fixed with paraformaldehyde (4% PFA in PBS), and the adipogenic differentiation was evaluated using Oil-Red staining.


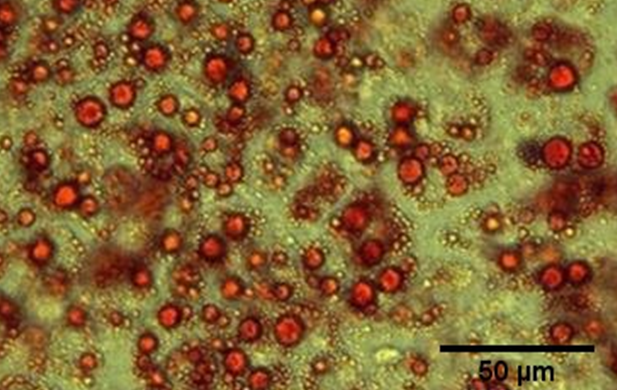


**Figure S3.** Oil-Red staining of ADSCs showing adipogenic differentiation of ADSCs

**Osteogenic Differentiation:**

To assess the osteogenic potential of adipose-derived stem cells (ADSCs), 2×10^4^ cells/cm² were plated in a 4-well cell culture plate. The Ham’s/F12 medium was supplemented with 100 nM dexamethasone, 50 μg/ml ascorbic acid, and 10 mM β-glycerol phosphate. After 21 days, the cells were fixed in 4% PFA and stained with 2% Alizarin Red S to determine calcium deposits.

**
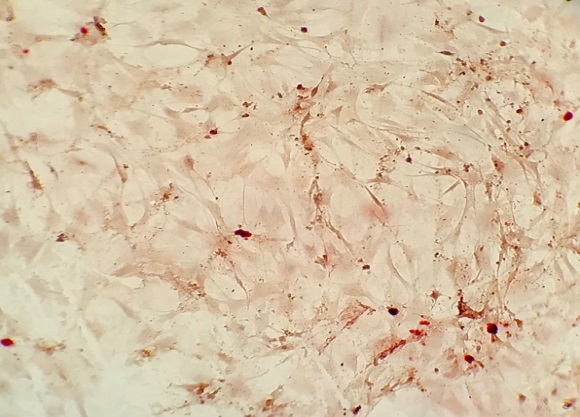
**

**Figure S4.** Alizarin Red staining of ADSCs showing osteogenic differentiation of ADSCs

**Chondrogenic Differentiation:**

The chondrogenic differentiation was conducted using a pellet culture system. A total of 3×10^5^ cells were centrifuged at 1400 rpm for 5 minutes. The supernatant was replaced with 1 ml of Ham’s/F12 medium containing 10 ng/ml TGF-β1, 50 μg/ml ascorbic acid, and 10^−4^ mM dexamethasone. After 21 days, the pellets were fixed in 4% PFA, and stained with Alcian Blue.


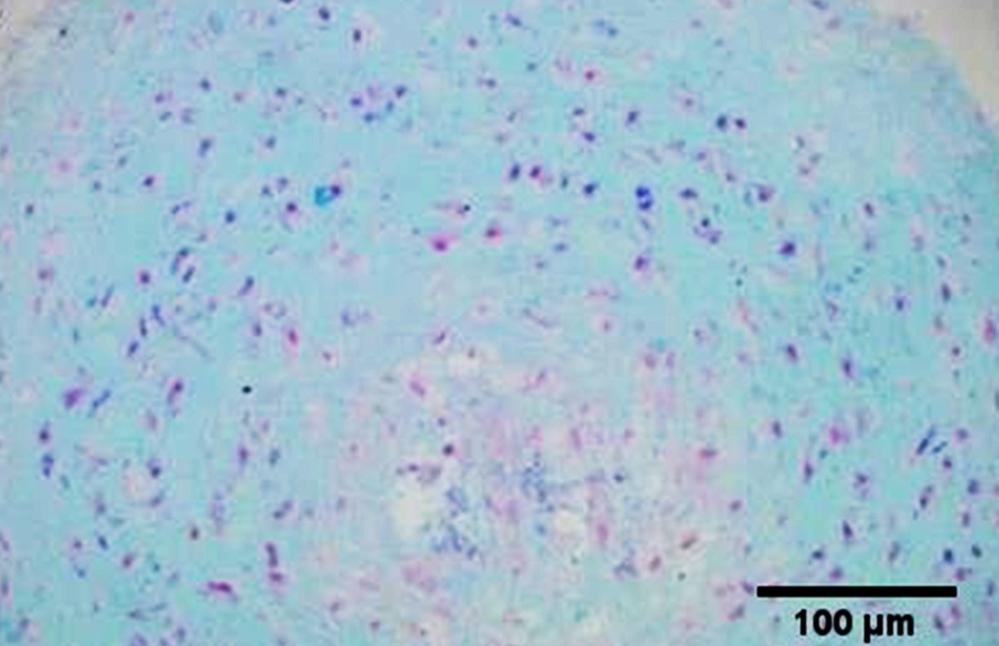


**Figure S5.** Alcian Blue staining of ADSCs showing chondrogenic differentiation of ADSCs
